# The influence of laser scribing on magnetic domain formation in grain oriented electrical steel visualized by directional neutron dark-field imaging

**DOI:** 10.1038/srep38307

**Published:** 2016-12-02

**Authors:** P. Rauscher, B. Betz, J. Hauptmann, A. Wetzig, E. Beyer, C. Grünzweig

**Affiliations:** 1 Laser Ablation and Cutting, Fraunhofer IWS, Dresden, Germany; 2 Laboratory for Neutron Scattering and Imaging, Paul Scherrer Institute, CH-5232 Villigen-PSI, Switzerland; 3 Institute of Manufacturing Technology, TU Dresden, Dresden, Germany

## Abstract

The performance and degree of efficiency of transformers are directly determined by the bulk magnetic properties of grain oriented electrical steel laminations. The core losses can be improved by post manufacturing methods, so-called domain refinement techniques. All these methods induce mechanical or thermal stress that refines the domain structure. The most commonly used technique is laser scribing due to the no-contact nature and the ease of integration in existing production systems. Here we show how directional neutron dark-field imaging allows visualizing the impact of laser scribing on the bulk and supplementary domain structure. In particular, we investigate the domain formation during magnetization of samples depending on laser treatment parameters such as laser energy and line distances. The directional dark-field imaging findings were quantitatively interpreted in the context with global magnetic hysteresis measurements. Especially we exploit the orientation sensitivity in the dark-field images to distinguish between different domain structures alignment and their relation to the laser scribing process.

Transformers made an important prerequisite for the use of electric energy and for technical industrial development of the last century. The losses in European Union for distribution transformers are estimated at about 33 TW · h/year^1^. The basic structure of the power transformer with components core, winding and insulation structure has not changed since that time. The transformer cores are made of stacked grain oriented (GO) electrical steel laminations. The losses in the GO laminations consist essentially of hysteresis and classic eddy current and anomalous eddy current losses. To reduce the classic eddy current losses the thickness of the GO laminations is reduced from 0.35 mm down to 0.18 mm however reaching a limit due to the fabrication process.

Concerning the other loss components, the laser scribing of GO electrical steel laminations is one method to increase the efficiency of power and distribution transformers. The theoretical approach is the reduction of the anomalous eddy current loss driven by demands for the efficient usage of energy. Thus manufacturing processes, like laser scribing, have been continually improved over the last decades^2^.

It is known that the laser irradiation induces residual stress that refines the domain structure, which influences the global magnetic properties^3^,^4^,^5^,^6^. Nevertheless, the fundamental influence to the bulk domain behavior is still not fully understood due to the lack of observation techniques. Surface domains can be observed using surface-sensitive magneto-optical Kerr effect (MOKE) or magnetic powder pattern, in which ferromagnetic particle from a colloidal suspension interact with the stray fields of the sample. Both methods are well established, but deliver only information of the surface domain structure^6^.

Neutron grating interferometry (nGI) is one approach to visualize bulk magnetic structures spatially resolved in the neutron dark-field image (DFI)^7^,^8^,^9^,^10^. This method has been used to investigate GO steel^11^,^12^,^13^,^14^,^15^ as well as non-grain oriented steel grades^16^, which demonstrates the general possibility to investigate bulk magnetic behavior of silicon steel sheet without any preparation (removing of the isolating coating layer) that has be shown recently influencing the domain structure^13^. In the following we present the observation of bulk and supplementary domain structures and the refinement of them of an untreated and differently laser treated high-permeability GO electrical steel laminations.

## Samples and experimental techniques

### Differently laser treated samples

For the experiment, four representative samples in the Epstein geometry of 300 mm × 30 mm × 0.27 mm high-permeability GO electrical steel (M110–27P according to DIN EN 10107) with an isolation layer consisting of a forsterite (Mg_2_SiO_4_) and phosphate layer were used. The samples were annealed after cutting into Epstein strips at 800 °C for 2 hours in a nitrogen atmosphere in order to perform stress-relief annealing.

The laser treatment was performed with the Laser Magnetic Domain Refinement (LMDR) test system installed and developed at the Fraunhofer IWS Dresden for industrial related material investigations of continuous moving material^17^. The LMDR test system was equipped with a continuous wave multi-mode fiber laser having a maximum output power of 4000 W. The laser spot on the surface of the steel sheets was elliptically shaped due to the optical configuration of the LMDR test system. The semi minor axis R_X_ was aligned along rolling direction (RD). The semi major axis R_Y_ was aligned along the scanning speed, perpendicular to the RD. Lateral spot velocities of up to 300 m/s can be applied with the LMDR test system in order to simulate industrial related material treatments.

For the investigation of the influence of the laser scribing parameters such as laser energy and line distance one untreated (sample 1) and three representatively laser treated samples (sample A, B, C) were selected. The different laser treatment parameters as well as their influence on the magnetic properties are summarized in Table 1. The samples were magnetically characterized measuring B-H curves before and after the laser scribing process in order to evaluate the change of the magnetic properties such as core loss reduction and change of polarization as shown in Table 1. For these measurements a 250 mm × 30 mm single sheet tester (SST) and a measurement device (MPG 200) from Brockhaus were used. An increase of the laser energy by a factor of 4 results in visible laser lines and thereby a damage of the surface coating as can be seen for the samples B and C.

**Figure 1:**
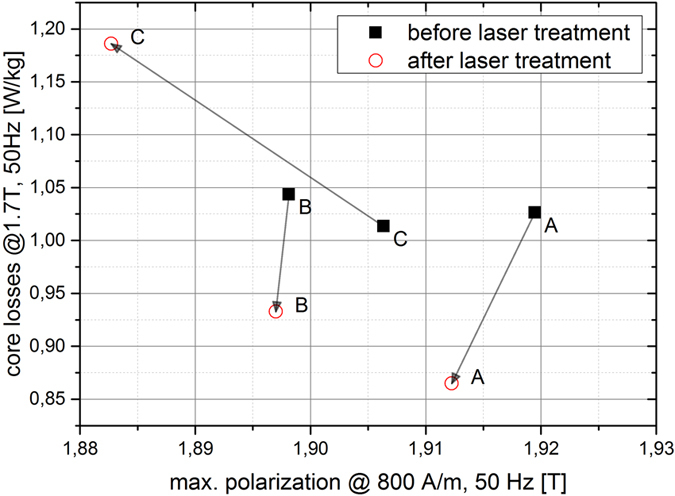
Absolute changes of the magnetic properties of for three different laser treated samples (A,B,C) before and after the laser treatment.

Even if the objective target of the laser treatment is the core loss reduction, one sample (sample C) with higher core losses after laser scribing was selected beside of two samples were the core loss was reduced (sample A, B). The absolute changes of the core losses and the maximum polarization are illustrated in Fig. 1 for all three laser treated samples (A, B, C). The black rectangle shows the values before the laser treatment, the red circles shows the values after laser treatment. The arrow indicates the changes of the magnetic values due to laser scribing process using different treatment parameters. It can be clearly seen that the core losses are reduced for sample A (from 1.03 W/kg to 0.87 W/kg) and B (form 1.04 W/kg to 0.93 W/kg) but increased for sample C (form 1.01 W/kg to 1.19 W/kg).

**Figure 2:**
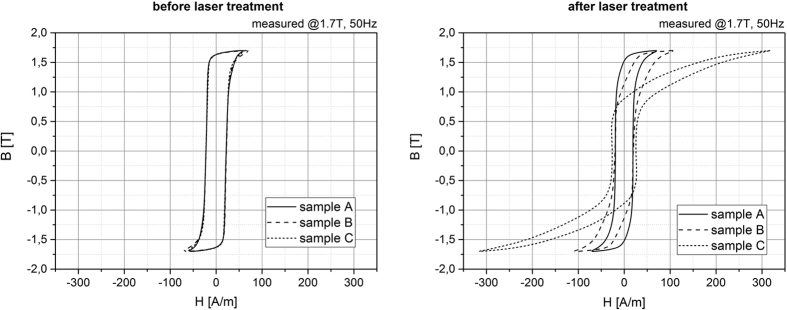
B-H curve measured at 1.7 T and 50 Hz (a) before laser treatment (left) after laser treatment (right) for the samples A, B and C.

The best results in terms of the core loss reduction as well as for the damage of the isolation layer are obtained for the treatment parameters used for sample A. However, the treatment parameters can be adapted by additional changing the line distance. A short line distance (sample C, d_L_ = 2 mm) leads to a deterioration of the core losses whereas an increase of the lines spacing (sample B, d_L_ = 10 mm) results in a core loss reduction. The B-H curves before and after the laser treatment, as show in Fig. 2, illustrate the magnetization behavior dependent on the laser treatment parameters. The highest shearing of the magnetic curve after laser treatment was observed for sample C with reduced magnetic properties. It can be seen that the coercive field strength is for sample C higher than for samples A and B due to the fact the B-H curve is wider at B = 0 T.

From Table 1 and Fig. 1 we can clearly see that the laser treatment of the GO laminations has an impact of the hysteretic behavior of the sample in the global B-H measurement. The physical nature of the differences in the B-H curve is given by changing the underlying magnetic domain formation during the laser treatment. However, the bulk and supplementary domain structure remain hidden in the global measurements. In the following we present how neutron grating interferometry providing the dark-field image delivers information about the change of the domain structure after the laser treatment. In particular we interpret and correlate these findings with the global B-H measurement results.

### Neutron grating interferometry

**Figure 3:**
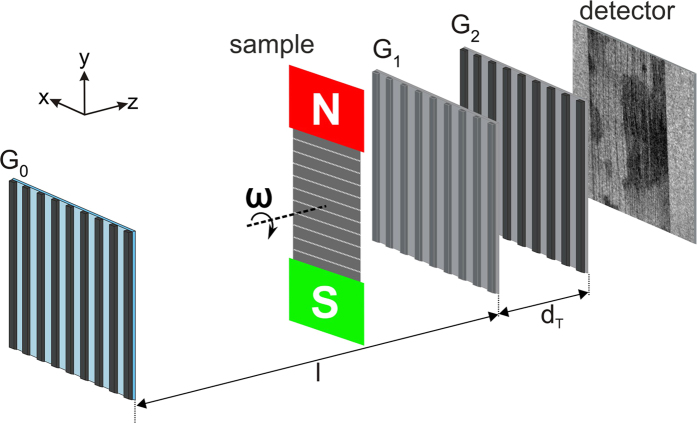
Schematic of the nGI setup with source grating G_0_, the phase grating G_1_ and the analyzer grating G_2_ and the magnetic sample environment to magnetize the laser treated GO steel lamination. The sample including magnetic field can be rotated around an axis perpendicular to the lamination around the angle ω to record the directional dark-field images.

The nGI experiments were performed at the Swiss Spallation Neutron Source (SINQ) at the Paul Scherrer Institut. The nGI setup consists of three gratings^18^, as schematically shown in Fig. 3. The use of the source grating *G*_*0*_ acting as a periodic line source, followed by the phase grating *G*_*1*_ at a distance *l* = 5,23 m, and the analyzer grating *G*_*2*_ at the Talbot distance *d*_*T*_ = 19,4 mm behind *G*_*1*_, allows for the spatially resolved detection of the samples scattering properties^13^. The interaction of the magnetic moment of a neutron with the magnetic induction of ferromagnetic domains results in a local scattering of the incoming neutron beam, which is visualized in the corresponding DFI. Further details about the contrast formation can be found in refs 7, 8. The DFIs were recorded by a conventional scintillator (50 μm thick ^6^LiF/ZnS) based detection system using a digital camera [Andor NEO sCMOS, 2160 × 2560 pixels, pixel size: 6.5 μm] placed behind *G*_*2*_. The effective spatial resolution in our measurements is 70 μm and is determined by intrinsic blurring of the scintillation screen^19^ and penumbra blurring caused by the sample to detector distance of 3 cm.

In the experiments the samples have been magnetized in a magnetic frame. The DFI images have been taken stepwise by increasing the magnetic field strength (DC magnetization) after demagnetization. The magnetization coils of the magnetic frame are supplied with currents up to 4 A using a power supply [KEPCO BOP100-4ML]. In order to distinguish between different alignments of the magnetic structures, the orientation of the sample with respect to the gratings could be varied by the angle ω. The ω = 0° orientation was defined when the magnetic field orientation is parallel to the grating lines, as can be seen in Fig. 3. A rotation of the magnetic frame of 90° around the beam axis of the neutrons leads to an orientation of the sample that is horizontal to the grating lines, which is defined as ω = 90° orientation. The magnetic field strength rotates with the rotation of the magnetic frame so that the magnetization vector is in both cases (ω = 0° and ω = 90°) parallel to the rolling direction of the lamination. The orientation dependency of the nGI setup was reported in ref. 7 for a small iron silicide single-crystal disk. Here we are using this dependency to observe the bulk magnetic domain structure in GO lamination (y direction for ω = 0°) and the supplementary domain structures in the laser lines (x direction for ω = 90°). As the laser lines are lying perpendicular to the magnetization direction we choose the two orientation of ω = 0° and ω = 90° to perform the direction dependent DFI experiments.

## Results and Discussion

### Directional dark-field images of the different laser treated samples

**Figure 4:**
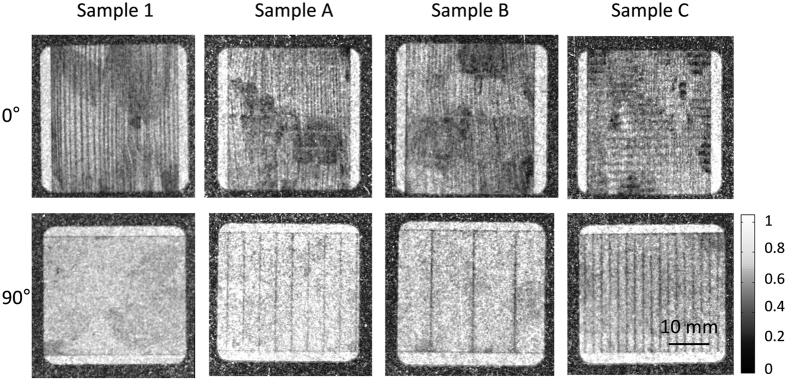
DFI images of the bulk and supplementary domain structures for the ω = 0° and ω = 90° (clockwise) orientation of the untreated sample 1 and the different laser treated samples (**A**–**C**). The data were taken without applied field (H = 0 A/m) after demagnetization. The laser lines and their distance of the treated samples A,B,C are clearly visible in the ω = 90° orientation as vertical black lines.

Figure 4 summarizes the DFI images for untreated and laser treated samples after demagnetization (H = 0 A/m) in ω = 0° and ω = 90° orientation taking advantage of the orientation dependent character of the DFI in context with domain wall formation. For the ω = 90° the sample was rotated clockwise by 90°. In the ω = 0° orientation (images in the top row) vertical black lines become visible, which are the volume domain walls spatially resolved. The smaller volume domain wall spacing of the laser treated samples A, B, C in comparison to the untreated sample 1 can be explained by the refinement of the basic domains due to laser scribing. The dark areas in the DFIs for the ω = 0° orientation are grains with a certain misorientation to the RD and hence forming supplementary surface domains^14^. These domains are below the detection limit and are therefore detected as darker areas.

The DFI images of the ω = 90° orientation show a significant difference in comparison to the untreated sample for the magnetic field strength of H = 0 A/m. In the ω = 90° orientation (images in the bottom row) the laser affected areas become visible as black vertical lines perpendicular to the RD. Thus we can observe the laser lines directly by the created domain structure. A more detailed investigation of the supplementary domains can be found in literature^20^ where scanning electron microscopy was used to observe the detailed domain structure without removing the isolation coating layer. The volume domain walls lying perpendicular to the grating lines and visible in DFIs for ω = 0° do not contribute to the DFI signal in the ω = 90° orientation. The pronounced vertical feature in the DFIs in the ω = 90° orientation are solely due to the supplementary domains formed in the laser lines. Hence we can obtain information about the orientation of the domain walls even if they are below the detector resolution. The applied line distances was increased from d_L_ = 2 mm (sample C) to d_L_ = 4 mm (sample A) and d_L_ = 10 mm (sample B), which can be observed in the corresponding DFIs. The dramatically changes of the observed bulk magnetic domains are associated with the Goss texture ({011} 〈100〉) and the preferred alignment of the domains along the RD. The domain structure in ω = 90° orientation being not visible can be explained by the fact that the magnetic domains of GO electrical steel are almost aligned in the RD. Domains with slight misorientation will be aligned parallel to the magnetic field strength during magnetization. A contribution of the DFI signal is not expected for the ω = 90° orientation because the occurring magnetic scattering is towards a direction, where the nGI is insensitive for the direction of the domain walls. Back in the DFIs in the ω = 0° the laser lines are only visible in the misoriented grains when they cross these areas and become visible as bright horizontal lines. In the following we use the direction dependent character of the DFI to visualize the domain structures of the non-treaded and different laser treated samples during magnetization.

### Domain formation during magnetization of the untreated sample

**Figure 5:**
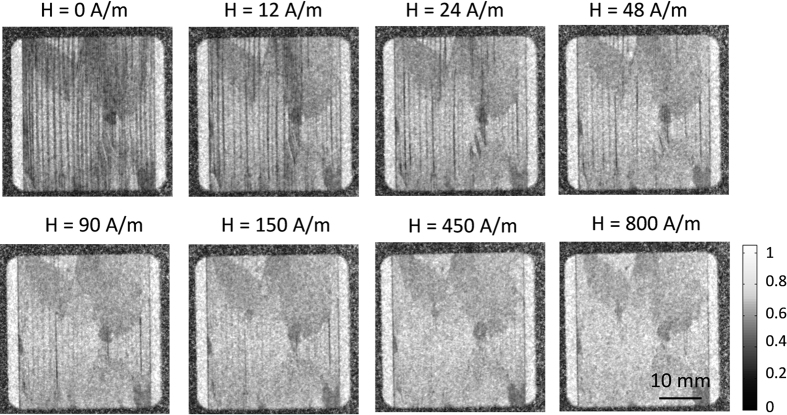
Directional DFIs of the untreated sample 1 at different magnetic field strengths for the orientation of ω = 0°. (magnetic field parallel to grating lines of the nGI setup).

The DFI images at different magnetic fields for the untreated samples (sample 1) are illustrated for the ω = 0° orientation in Fig. 5. The bulk magnetic domains are visible spatially resolved as black vertical lines parallel to the RD representing the volume domain walls. The first image (H = 0 A/m) was taken after demagnetization. Afterwards the magnetic field strength was increased stepwise until H = 800 A/m. The domain structure resolved as vertical lines vanishes while increasing the magnetic field strength until no volume domain structures are visible at about H = 450 A/m. The most significant changes of the domain walls was found between H = 12 A/m and H = 48 A/m. At higher magnetic fields of H = 90 A/m and H = 150 A/m vertical lines are weakly visible in the DFI images. Areas with a reduced DFI signal were observed for all applied magnetic field strength, illustrated as slightly darker regions as can be seen in the upper left or upper right corner of the DFI images in Fig. 5. These areas show only a minor reaction on the applied magnetic field. These regions are identified to misoriented grains exhibiting supplementary domain structures.

The general interpretation of the DFI signal in relation to the magnetic domain structure was discussed in refs 7, 8, 9, 10, 11, 12, 13, 14, 15. The spatially resolved magnetic structures illustrated as black vertical lines parallel to the RD can be attributed to domain walls of 180° basic domains. In agreement with the theoretical consideration for the behavior of the magnetic domains, it can be stated the amount of the domains walls decrease while increasing the magnetic field strength, which can be observed in the DFI images in Fig. 5.

**Figure 6:**
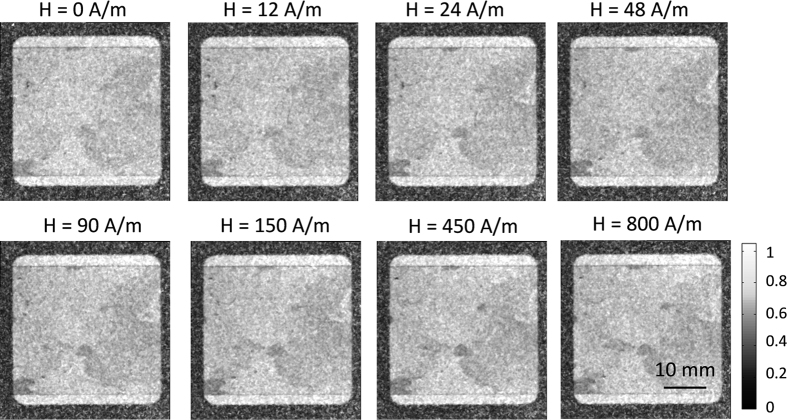
Directional DFIs of the untreated sample 1 at different magnetic field strengths for the orientation of ω = 90°. (magnetic field perpendicular to grating lines of the nGI setup).

In case of the orientation ω = 90° the DFI images for different magnetic field strength are illustrated for sample 1 in Fig. 6. The domain walls, which are visible for the ω = 0° orientation, could not be observed during magnetization of the ω = 90° orientation due to the direction dependent nature of the DFI. Contrasts in the DFI images, like in the lower left-hand corner, are caused by misoriented grains. The same structure was observed in the lower right-hand corner for the ω = 0° orientation (see Fig. 5). The DFI images of the untreated GO electrical steel in ω = 0° and ω = 90° orientation has shown that the alignment of the sample with respect to the grating lines has to be considered during magnetization and can be used to differentiate between 0° and 90° orientations of bulk and supplementary domains.

### Domain formation during magnetization of laser treated samples

**Figure 7:**
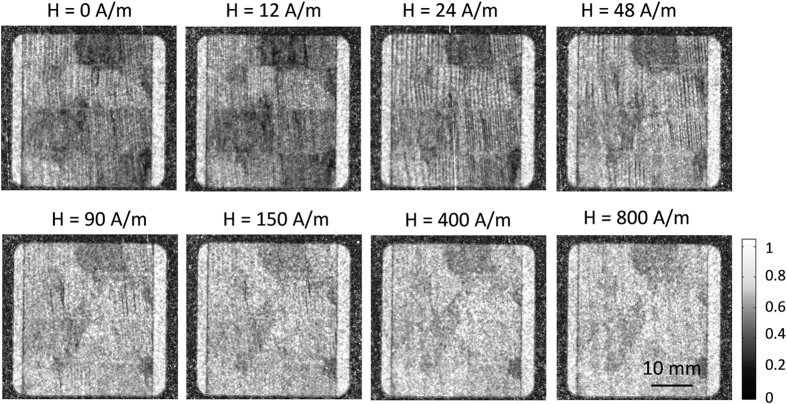
Directional DFIs of the laser treated sample B at different magnetic field strengths for the orientation of ω = 0° (magnetic field parallel to grating lines of the nGI setup).

**Figure 8:**
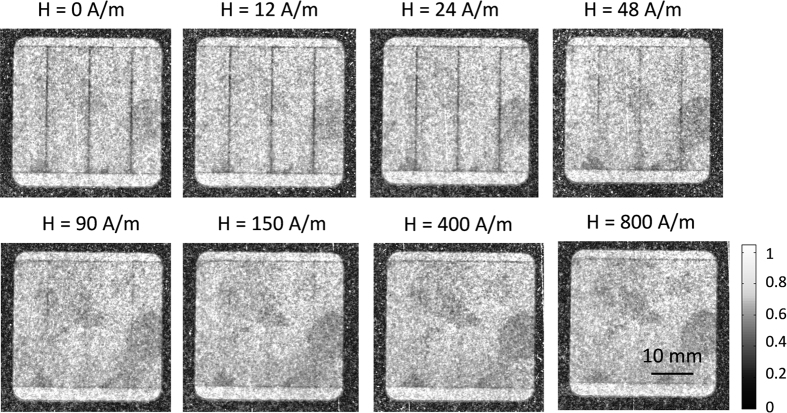
Directional DFIs of the laser treated sample B at different magnetic field strengths for the orientation of ω = 90° (magnetic field perpendicular to grating lines of the nGI setup).

The laser treated samples have been similarly investigated at the different magnetic fields as the untreated in Fig. 6. Representatively for laser treated samples we choose sample B to illustrate the domain wall formation during magnetization. The directional DFI results for sample B are shown in Fig. 7 for the ω = 0° orientation and in Fig. 8 for ω = 90° orientation. The corresponding directional DFI data for sample A and C can be found in the supplementary material (Fig. S1-S4). For the orientation ω = 0°, domain structures are spatially resolved as black vertical lines similar to the untreated samples 1, while the black vertical lines themselves are again the volume domain walls. It is clearly visible that the laser scribing leads to a refinement of the volume domains. Hence, after demagnetization the quantity of the volume domain walls increases at H = 12 A/m. Afterwards, the domain walls vanish until no walls are visible at about H = 450 A/m. Regions where the magnetic structure cannot be spatially resolved are darker, as can be seen for example at the middle upper position in Fig. 7. The distances of the domain walls of the basic domains are in general smaller in comparison to the untreated sample, which is expected due to the refining of the basic domains after laser scribing.

If the sample is rotated 90° clockwise around the beam axis, the observed DFI signal changes significantly as shown in Fig. 8. The previously observed domain walls of the 180° basic domains cannot be found in the DFI images due to the parallel orientation of the magnetic domain walls to the grating lines of the nGI setup similar to the untreated sample. However, now the domains walls related to the laser affected zone become visible. They are visualized as black lines pointing in the direction perpendicular to the RD. Note, this are not the basic domains. The laser lines appear visible as supplementary domains are formed within the laser line with a 90° orientation perpendicular to volume domains and to the RD. This “created” supplementary domain structure vanishes by increasing the magnetic field strength. Interesting, at about 150 A/m, the supplementary domains related to the laser lines cannot be observed in the DFI images in case of sample B. Darker regions in the DFI images, like in the middle right position, are slightly influenced by magnetic field strength and still visible at higher magnetic field strengths.

In the following we will correlate the direction dependent domain formation as observed locally in the DFI with global B-H measurements.

### Interpretation and correlation of the directional DFI signal to global magnetic measurements

An existing approach to correlate the DFI signal to global magnetic measurements was given for non-grain oriented (NGO) electrical steel^16^. However, it must be considered that NGO steel distinguishes itself considerably from GO electrical steel by a number of facts, like the grain size and orientation. This results in a different magnetization process and domain structure. The influence of the orientation of the sample to the grating lines of the nGI setup has to be considered and can be exploited in our case for the domain formation in GO laminations. The DFI images of the untreated sample have shown that almost most significant information’s of the magnetic structures are given for a ω = 0° orientation where the RD and the magnetic field are parallel to the grating lines in comparison to the laser treated samples. For the quantitative assessment of the directional DFI signals in relation to the laser treatment parameter under consideration of the measured B-H curve two new different approaches are used.

For the following comparison between the global B-H and the local DFI results a global or average DFI signal was defined in order to quantify the nGI measurement dependent on the magnetic field strength as follows


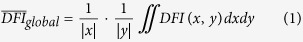


with *x* defined as direction perpendicular to RD and *y* defined as direction in RD. The average global DFI signal is used to quantify the general magnetic behavior during the magnetization process. With mathematical description of the global DFI signal in relation to magnetic flux in Eq. 1 we are able to quantitatively evaluate the relative change depending on the laser treatment parameters and the magnetic field strength. Beside the global description of the DFI signal an additional a local DFI signal was defined in order to quantify the general magnetic behavior of the domain structure inside the laser affected area and given by the equation


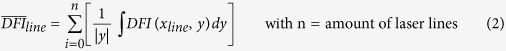


applied for the DFI images in ω = 90° orientation. Hereby we can describe the influence of the laser irradiation on the magnetic structure even if the magnetic domains cannot be spatially resolved.

**Figure 9:**
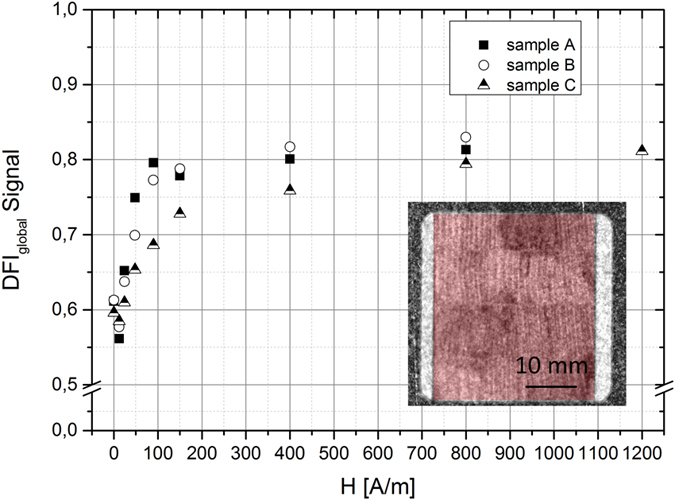
Global DFI signal of laser treated samples (A, B, C) measured for ω = 0° orientation with increasing magnetic field strength (area marked by red indicates the boundaries for the calculation).

The global DFI_global_ signal as function of the applied magnetic field strengths is plotted for the laser treated samples A,B,C in ω = 0° orientation in Fig. 9. The red marked area in the diagram indicates the area that is used for the calculation. After demagnetization a global DFI_global_ signal of about 0.6 was measured for the laser treated samples. For the first applied magnetic field strength (H = 12 A/m) a reduced DFI_global_ signal was observed for all sample. This can also be observed in the DFI images, in which the amount of the spatially resolved domain walls is slightly increased. Afterwards, the DFI_global_ signal increases for all laser treated samples. It is noticeable that the samples (sample A, B) with reduced core losses after laser scribing have steeper gradients in comparison to the sample (sample C) with deteriorated magnetic properties. The maximum DFI_global_ signal of 0.8 was obtained for sample A and B at a magnetic field strength of H = 400 A/m. In contrast, sample C reaches the maximum DFI signal at about H = 800 A/M. The different DFI_global_ curves of each sample indicate a different magnetization process that can be correlated to the B-H curve after laser scribing as seen in Fig. 2. A higher magnetic shearing of the B-H curve results in a smaller increase of the DFI_global_ signal while increasing the magnetic field strength. The general behavior of the global DFI signal is in good agreement with the theoretical understanding of domain wall formation during magnetization, where an increase of the magnetic field strength leads to a reduced amount of domain walls and therewith an increase of the DFI_global_ signal.

**Figure 10:**
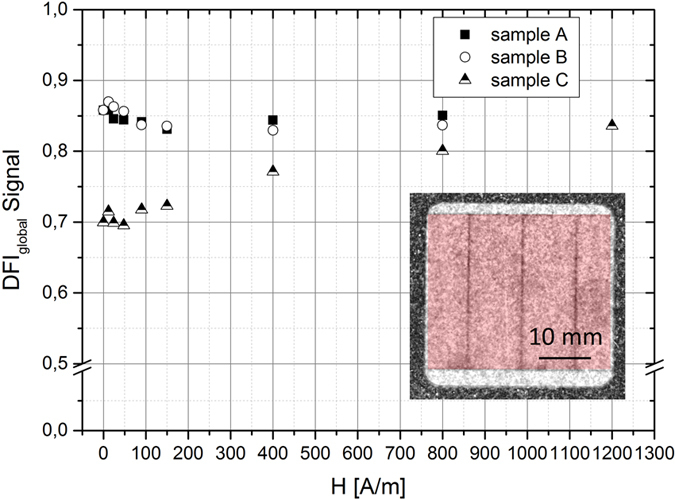
Global average DFI signal of laser treated samples (A, B, C) measured in ω = 90° orientation with increasing magnetic field strength (area marked by red indicates the area used for the calculation).

The global average DFI_global_ signal as function of the applied magnetic field strength for the ω = 90° orientation are shown for all three laser treated samples A,B,C in Fig. 10. Almost no change of the global DFI_global_ signal (DFI_global_ ≈ 0.85) was observed while increasing the magnetic field strength for the samples A and B that have be improved by laser scribing. Sample C with deteriorated core losses due to the laser process shows a different behavior. The global DFI_global_ signal after demagnetization (H = 0 A/m) is lower (DFI_global_ ≈ 0.7) than the DFI_global_ signals for sample A and B and increase slightly while increasing the magnetic field strength until a maximum of about 0.84 at H = 1200 A/m. However, the absolute DFI_global_ values of sample C are below the DFI_global_ values of sample A and B for all applied magnetic field strengths. This means that even if the laser lines in the DFI images in ω = 90° orientation vanishes for all samples at the same magnetic field strength of about H = 150 A/m, the amount of domains structures seems to be higher for samples C in comparison to sample A and B due to the reduced DFI signal (see Fig. 10).

**Figure 11:**
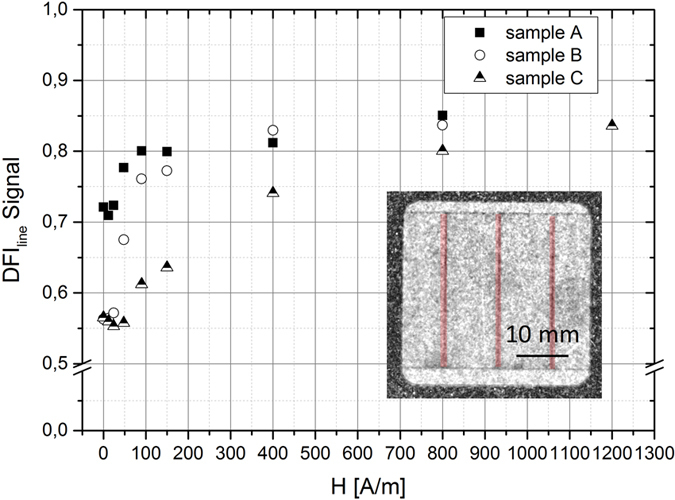
Local DFI signal of laser treated samples measured in ω = 90° orientation with increasing magnetic field strength (area marked by red indicates the area used for the calculation).

The local DFI_line_ signal in ω = 90° orientation, which has been used to evaluate the domain structure inside the laser trace, is illustrated in Fig. 11. In contrast to the global average DFI signal in Fig. 10 only the red marked areas are considered for the calculation. The DFI_line_ signal of the laser line increased during magnetization process, which means that the domains wall density inside that area is reduced while increasing the magnetic field strength. It is noticeable, that the DFI_line_ signal after demagnetization is different dependent on the used laser energy, which is defined as laser power multiplied with dwell time. Sample A, which has been laser scribed with energy of 51 mJ, has a significant higher DFI value (DFI_line_ ≈ 0.72) after demagnetization than the samples B (DFI_line_ ≈ 0.56) and C (DFI_line_ ≈ 0.56), which have been laser treated with 204 mJ. After demagnetization a slightly reduced local DFI_line_ signal was measured for the first applied magnetic field strengths. Afterwards, the DFI_line_ values increases while increasing the magnetic field strength until the maximum of about 0.85 is reached for all samples. Under consideration of the treatment parameters, it can be stated that the samples treated with the same energy (sample B and C) of 204 mJ have almost the same DFI_line_ signals after demagnetization, but show a different behavior during the magnetization process. Sample B, where the core losses have been reduced, has a steeper gradient than sample C, with an increase of the core losses after the laser treatment. For higher magnetic field strengths at about H = 400 A/m, the DFI_line_ values of sample A and B are comparable. It is assumed that the different DFI_line_ curves between samples B and C can be attributed to the different magnetization behavior as a result of the changed line distance, which has been reduced from 10 mm to 2 mm. The modification of the line distance results in different B-H curves and core losses, which are fundamentally related to the domain structure. The reduced local DFI signal as shown in Fig. 10 in dependence of the applied laser energy is in agreement with the effect discussed in ref. 21 where the width of the closure domains increase while increasing the local stress.

## Conclusion

The bulk magnetic domain behavior of four GO electrical steel sheets was investigated by neutron grating interferometry. The DFI signal was analyzed directionally with respect to the global magnetic measurements of one untreated and three representative laser scribed samples using different line distances of 2,4,10 mm and energies of 51 mJ and 204 mJ. We showed that the samples have to be investigated under ω = 0° and ω = 90° orientation to account for the contribution to the DFI signal of both the basic volume and supplementary domain structures. We were able to visualize the laser induced changes of the domain structure, especially around the laser affected area. Additionally, the DFI signal was discussed and quantitatively evaluated with respect to the different treatment parameters. We provide a novel evaluation approach in order to interpret the local DFI signal findings in correlation to the global magnetic measurements. Especially the local DFI_line_ signal for the ω = 90° orientation of the laser line shows a dependency to the used treatment parameters. In addition, the influence of laser irradiation on laser treated samples with invisible lines could be proven by the DFI images taken under 90 ° orientations. Our results show that the first quantitative approach to locally characterize the influence of laser scribed GO laminations by correlating of the DFI signal with respect to the magnetic properties is possible. Further investigations are needed to correlate the DFI signal with the magnetic induction values as obtained in a B-H measurement.

## Additional Information

**How to cite this article**: Rauscher, P. *et al*. The influence of laser scribing on magnetic domain formation in grain oriented electrical steel visualized by directional neutron dark-field imaging. *Sci. Rep.* **6**, 38307; doi: 10.1038/srep38307 (2016).

**Publisher’s note:** Springer Nature remains neutral with regard to jurisdictional claims in published maps and institutional affiliations.

## Supplementary Material

Supplementary Matieral

## Figures and Tables

**Table 1:** Three different laser treated samples A, B and C regarding laser energy and laser line distance.

Sample	Laser energy	Line distance	Core loss reduction	Change of Polarization	Coating Damage
	mJ	mm	W/kg	%	
A	51	4	15,7%	−0,4%	no damage, invisible lines
B	204	10	10,6%	−0,0%	damage, lines visible
C	204	2	−17,0%	−1,2%	damage, lines visible

Global measurements provide the relative core loss reduction (measured at 50 Hz, 1.7 T) and change of polarization (measured at 50 Hz, 800 A/m). The coating damage for the three samples is provided.
