# Supplementary material for: The influence of laser scribing on magnetic domain formation in grain oriented electrical steel visualized by directional neutron dark-field imaging
Source: Sci Rep. 2016 Dec 2;6:38307. doi: 10.1038/srep38307 (PMC5133608; doi:10.1038/srep38307)
Supplement: Supplementary Matieral [file srep38307-s1.pdf]

The influence of laser scribing on magnetic domain formation in grain oriented electrical steel visualized by directional neutron dark-field imaging

Authors

P. Rauscher[1], B. Betz[2], J. Hauptmann[1], A. Wetzig[1], E. Beyer[1,3], and C. Grünzweig[2,*]

[1] Laser Ablation and Cutting, Fraunhofer IWS, Dresden, Germany

[2] Laboratory for Neutron Scattering and Imaging, Paul Scherrer Institute, CH-5232 Villigen-PSI, Switzerland

[3] Institute of Manufacturing Technology, TU Dresden, Dresden, Germany

* Corresponding author: christian.gruenzweig@psi.ch

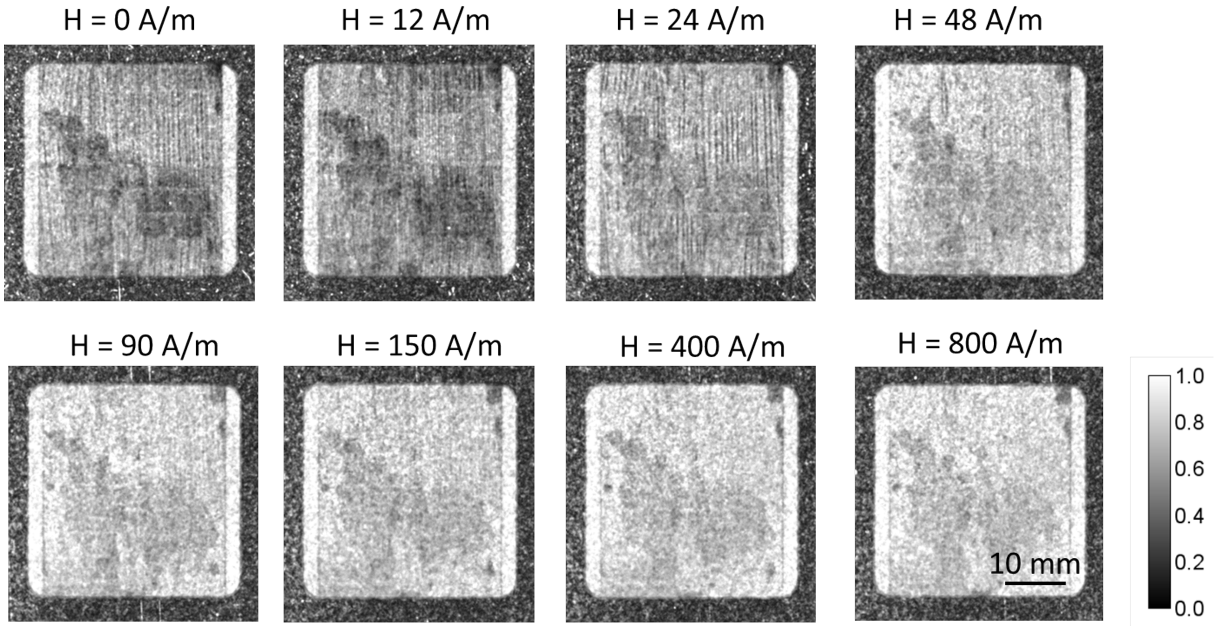

Figure S1: Directional DFIs of the laser treated sample A at different magnetic field strengths for the orientation of ω=0° (magnetic field parallel to grating lines of the nGI setup).

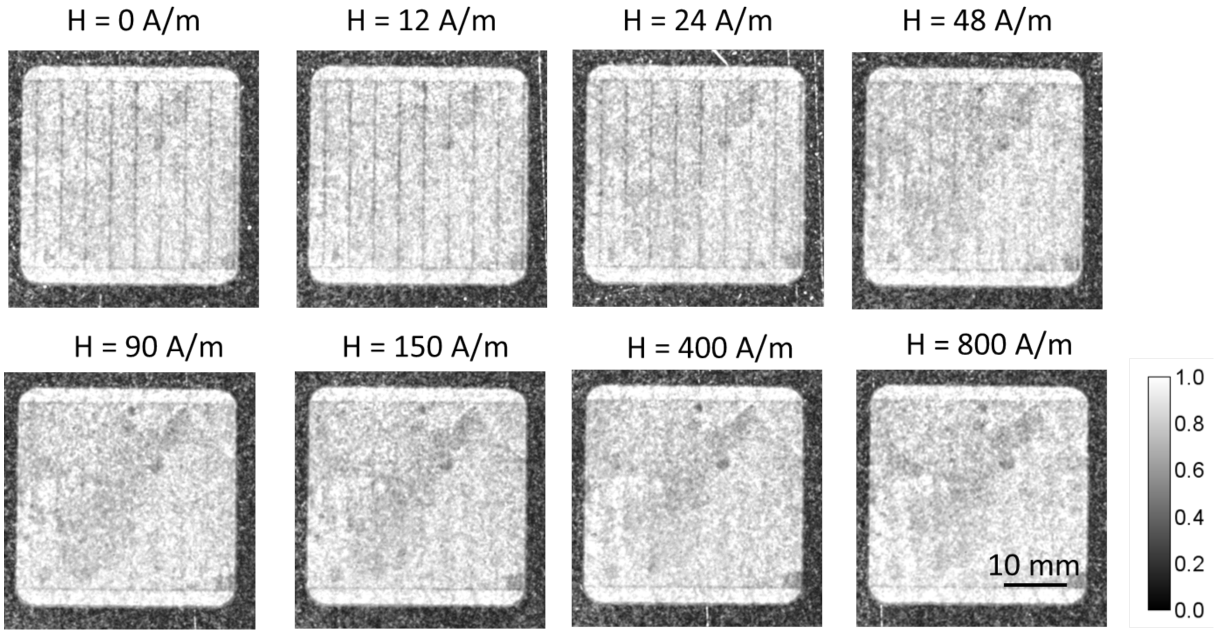

Figure S2: Directional DFIs of the laser treated sample A at different magnetic field strengths for the orientation of ω=90° (magnetic field parallel to grating lines of the nGI setup).

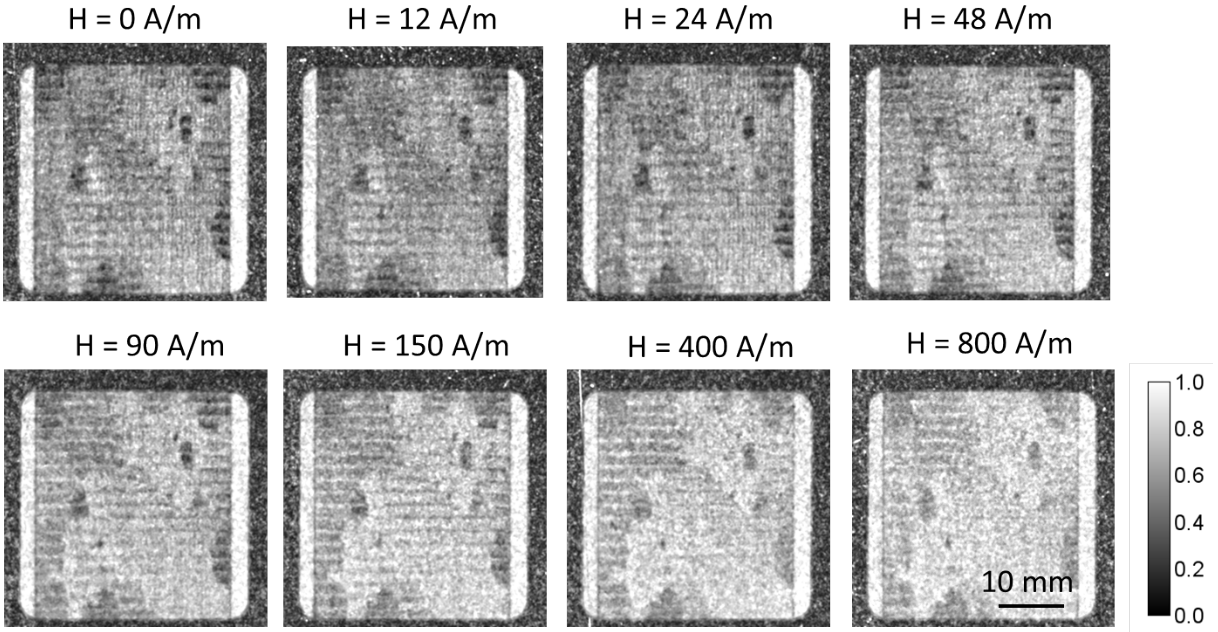

Figure S3: Directional DFIs of the laser treated sample C at different magnetic field strengths for the orientation of ω=0° (magnetic field parallel to grating lines of the nGI setup).

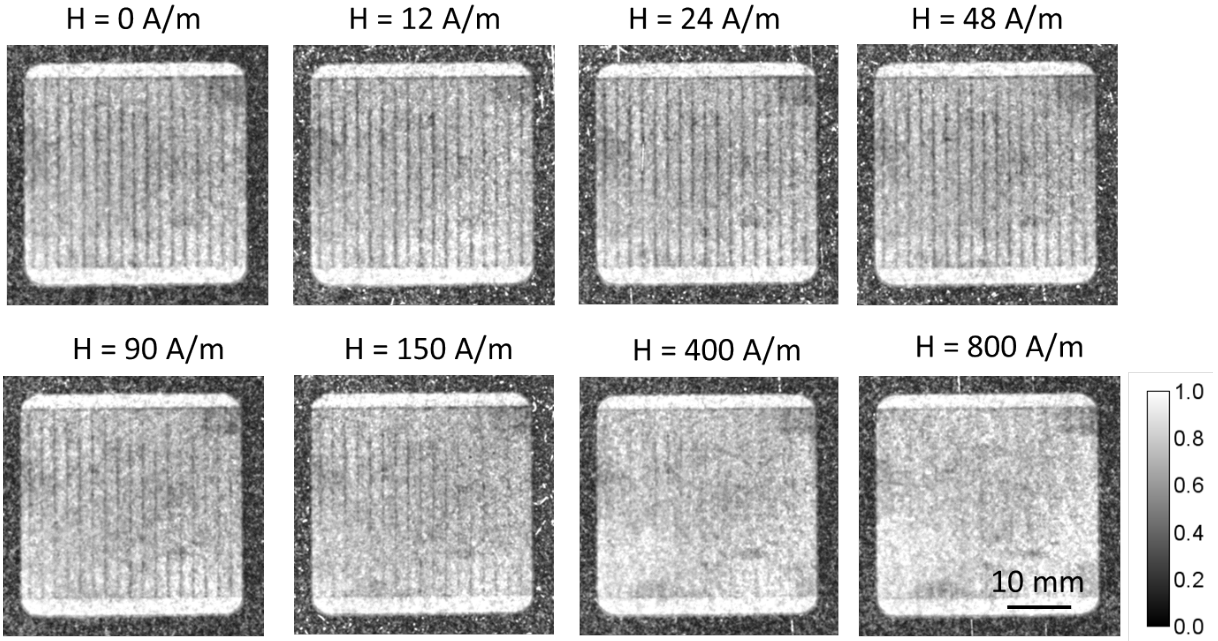

Figure S4: Directional DFIs of the laser treated sample C at different magnetic field strengths for the orientation of ω=90° (magnetic field parallel to grating lines of the nGI setup).
